# Horizontal Gene Transfer: From Evolutionary Flexibility to Disease Progression

**DOI:** 10.3389/fcell.2020.00229

**Published:** 2020-05-19

**Authors:** Melissa Emamalipour, Khaled Seidi, Sepideh Zununi Vahed, Ali Jahanban-Esfahlan, Mehdi Jaymand, Hasan Majdi, Zohreh Amoozgar, L. T. Chitkushev, Tahereh Javaheri, Rana Jahanban-Esfahlan, Peyman Zare

**Affiliations:** ^1^Drug Applied Research Center, Tabriz University of Medical Sciences, Tabriz, Iran; ^2^Biotechnology Research Center, Tabriz University of Medical Sciences, Tabriz, Iran; ^3^Kidney Research Center, Tabriz University of Medical Sciences, Tabriz, Iran; ^4^Stem Cell Research Center, Tabriz University of Medical Sciences, Tabriz, Iran; ^5^Nano Drug Delivery Research Center, Health Technology Institute, Kermanshah University of Medical Sciences, Kermanshah, Iran; ^6^Department of Medical Nanotechnology, Faculty of Advanced Medical Sciences, Tabriz University of Medical Sciences, Tabriz, Iran; ^7^Edwin L. Steele Laboratories, Department of Radiation Oncology, Massachusetts General Hospital and Harvard Medical School, Boston, MA, United States; ^8^Department of Computer Science, Metropolitan College, Boston University, Boston, MA, United States; ^9^Health Informatics Lab, Metropolitan College, Boston University, Boston, MA, United States; ^10^Department of Medical Biotechnology, School of Advanced Medical Sciences, Tabriz University of Medical Sciences, Tabriz, Iran; ^11^Faculty of Medicine, Cardinal Stefan Wyszyński University in Warsaw, Warsaw, Poland; ^12^Dioscuri Center of Chromatin Biology and Epigenomics, Nencki Institute of Experimental Biology, Polish Academy of Sciences, Warsaw, Poland

**Keywords:** horizontal gene transfer, antibiotic resistance, cancer, evolution, cell-free DNA, apoptotic bodies, exosomes, transposable elements

## Abstract

Flexibility in the exchange of genetic material takes place between different organisms of the same or different species. This phenomenon is known to play a key role in the genetic, physiological, and ecological performance of the host. Exchange of genetic materials can cause both beneficial and/or adverse biological consequences. Horizontal gene transfer (HGT) or lateral gene transfer (LGT) as a general mechanism leads to biodiversity and biological innovations in nature. HGT mediators are one of the genetic engineering tools used for selective introduction of desired changes in the genome for gene/cell therapy purposes. HGT, however, is crucial in development, emergence, and recurrence of various human-related diseases, such as cancer, genetic-, metabolic-, and neurodegenerative disorders and can negatively affect the therapeutic outcome by promoting resistant forms or disrupting the performance of genome editing toolkits. Because of the importance of HGT and its vital physio- and pathological roles, here the variety of HGT mechanisms are reviewed, ranging from extracellular vesicles (EVs) and nanotubes in prokaryotes to cell-free DNA and apoptotic bodies in eukaryotes. Next, we argue that HGT plays a role both in the development of useful features and in pathological states associated with emerging and recurrent forms of the disease. A better understanding of the different HGT mediators and their genome-altering effects/potentials may pave the way for the development of more effective therapeutic and diagnostic regimes.

## Introduction

The mobility of genetic information between different organisms, known as horizontal gene transfer (HGT), is a dynamic and persistent phenomenon that can have immediate or delayed effects in the recipient host (Husnik and McCutcheon, 2018). Although HGT is more common among bacteria-to-bacteria (mainly in groups of Archaea and bacteria), it also occurs between other organisms, such as bacteria that commonly serve as donors and organisms like fungi, plants, and animals which act as recipients (Garcia-Vallvé et al., 2000; Rancurel et al., 2017).

In 2015, Crisp et al. (2015) reported that their experiments and phylogenetic analysis prove that 145 foreign genes have entered into the human genome over the evolutionary period. Two years later, Salzberg (2017) showed that the human genome has incorporated only mitochondrial foreign genes and retroviral vectors. It was proven that HGT is involved in bacteria genome evolution, genome damage prevention, antibiotic resistance, virulence, and compatibility to environmental stresses (Hall et al., 2017). HGT usually occurs between taxa that are located in a closed and centralized environment. The possibility of HGT occurrence increases due to the proximal contact of donor-recipient genomes with each other (Adato et al., 2015; Brown and Wernegreen, 2019). The results of phylogenetic and eukaryotic nuclear genome analysis showed that the information expressed by HGT can affect a wide variety of genes (Baquero et al., 2019; Campos et al., 2019; Leclerc et al., 2019). Accordingly, HGT is essential for the evolution of both prokaryotic and eukaryotic genomes.

Transposable elements (TEs), as a typical example of HGT mediators from viruses to eukaryotes, have enormous implications in the evolution of the human genome (Deniz et al., 2019). LINEs (L1) as the only active TEs in the human genome are involved in the development and progression of various human diseases by activating otherwise silent promoters and/or promoting the deregulated expression of oncogenes and tumor suppressor genes (Briggs et al., 2018; Ardeljan et al., 2020). Besides cancer, TEs are also implicated in the pathogenesis of various well-known genetic- (Payer and Burns, 2019), metabolic- (Turcot et al., 2012; Costello and Schones, 2018; Castellano-Castillo et al., 2019), and neurological (Saleh et al., 2019; Tam et al., 2019) diseases. Moreover, LINE insertion which promotes genomic instability may pose a threat to the biosafety of stem cells which serve as valuable resources for regenerative medicine and cell-based therapies (Schumann et al., 2019). Importantly, recent data indicates that the mammalian genome has undergone a HGT related to exosomes that can affect the therapeutic efficacy of modern genome editing tools, such as clustered regularly interspaced short palindromic repeat (CRISPR) (Ono et al., 2019). Also, newly discovered HGT mediators in mammalian cells including vesicular particles (EVs) (Willms et al., 2018; Zhang et al., 2019), apoptotic bodies (Caruso and Poon, 2018; Xu et al., 2019; Battistelli and Falcieri, 2020), and cell-free DNA (cfDNA) are involved in different stages of cancer development and progression, as well as anti-cancer drug resistance and therapy failure. A detailed review of the implication of these newly discovered mediators in cancer is reviewed in our recent work (Baghban et al., 2020).

Here, various mediators of HGT, from HGTs discovered earlier to the newly discovered ones, are reviewed. An in-depth understanding of the causes and consequences of HGT would open new horizons for better management of the diversity of human genetic/metabolic/neurodegenerative diseases and may benefit translational medicine through more effective diagnostic and therapeutic options. Therefore, we explained the effects of HGT mediators, ranging from genome flexibility and the emergence of useful traits to the development of various health-related diseases, antibiotic/cancer drug resistance, and metastasis in the following sections.

## Causes (Mediators) of HGT

### HGT Mechanisms in Prokaryotes

**FIGURE 1:**
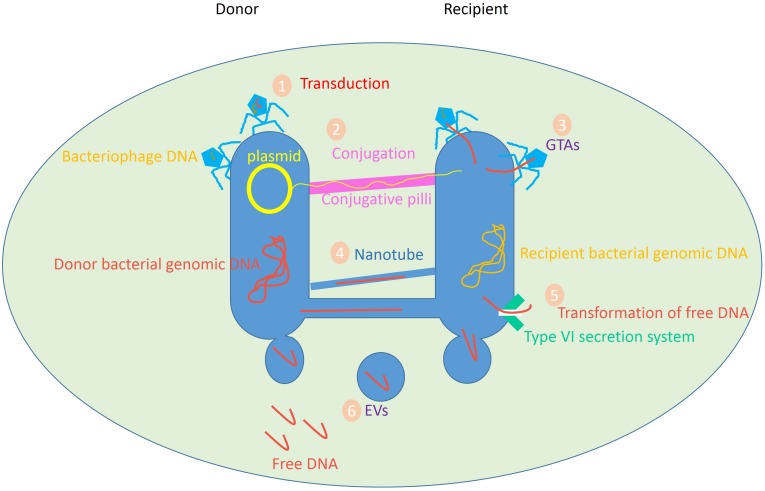
Variety of HGT mediators in prokaryotes. Data obtained from Ficht (2011), Lang et al. (2012), Borgeaud et al. (2015), Hall et al. (2017), Tzipilevich et al. (2017).

In nature, transformation, transduction, and conjugation are the principal mechanisms of HGT. Other mechanisms involve gene transfer agents (GTAs), nanotubes, and membrane vesicles (MVs) [which are also called extracellular vesicles (EVs) or exosomes] (Figure 1; Dauros Singorenko et al., 2017; Hong et al., 2019). Accordingly, the production of recombinant DNA can be achieved by directly transforming DNA or RNA from a donor, and subsequent integrating of foreign genetic material into the recipient cell’s genome. This phenomenon takes place in a wide variety of bacteria and is responsible for the transfer of mobile genetic elements (MGEs) such as transposons, integrons, and/or gene cassettes between bacterial species.

Meanwhile, as natural cloning systems and gene expression vectors, integrons are employed as an independent mechanism for gene transfer between many bacterial genomes, enabling recipients to acquire, carry, and express the antibiotic-resistant gene cassettes (Gillings, 2014). Of note, horizontal integron transfer is known to be the most important way for dissemination and diffusion of antibiotic resistance genes (ARGs) between bacterial strains. In addition, the capability of selective transfer of resistant genes promotes bacteria genome evolution to afford adaptation to environmental changes (Mazel, 2006; Engelstädter et al., 2016).

Likewise, transduction occurs when the bacteriophage carries a portion of the bacterial genetic information from a bacterium to another bacterium. In the process of conjugation, a complete DNA sequence, e.g., a plasmid, is transmitted between bacterial cells via direct cell-to-cell connection by conjugating pilus, which is proven to be an efficient process for the transfer of genetic material (Lang et al., 2012).

Gene transfer agents are bacteriophage-like particles known as natural vectors. Gene exchange through GTA was demonstrated for the first time in the purple non-sulfur bacteria Rhodobacter. Some bacteria produce GTAs to convey random portions of the host bacteria’s DNA to a receptor cell. Gene transfer by nanotubes and exosomes are the newly spotted mediators of HGT, where the transfer of genetic material goes beyond DNA (Bronkhorst et al., 2019).

Intercellular finite-sized nanotubes composed of bacterial-like membranes can bridge adjacent cells to readily exchange intracellular molecules including metabolites, protein, mRNA, and plasmid DNA. The mechanism of transfer involves the generation of a network of tubular conduits, enabling the transfer of the cytoplasmic content (Ficht, 2011). MVs, as bilayer structures, are shown to transfer biomolecules between bacteria in a protected form, so the likelihood of the successful gene transfer is high (Dubey and Ben-Yehuda, 2011; Brimacombe et al., 2015; Soucy et al., 2015; Shin et al., 2016; Domingues and Nielsen, 2017).

### HGT Mechanisms in Eukaryotes

Horizontal gene transfer occurs in various ways in multi-cellular organisms. For example, in plants, HGT can operate via natural factors, such as host-parasite connection. The parasite acts as a vector transferring mitochondrial genes among two diverse plant species. In fact, epiphytes and parasites can cause genetic changes as they transit DNA between plants (Richardson and Palmer, 2006).

Horizontal gene transfer via transposons is a prevalent method among both plants and animals that share genetic material. The transmission of transposons between rice and millet plant species is one of the clearest instances of genetic exchanges mediated by transposons, which are also known as jumping genes or selfish DNA (El Baidouri et al., 2014).

Other newly emerged mediators of HGT in eukaryotes with clinical implications include exosomes, apoptotic bodies, and cfDNA, which will be discussed in the next sections.

## Consequences of HGT: From Evolutionary Flexibility to Disease Progression

In the following sections, we overview the consequences of HGT from eliciting beneficial effects to causing challenging health-related diseases that occur by the genetic exchange among prokaryote-prokaryote, prokaryote-eukaryote, virus-eukaryote, and eukaryote-eukaryote, among others.

### Prokaryote-Prokaryote Genetic Exchange

**FIGURE 2:**
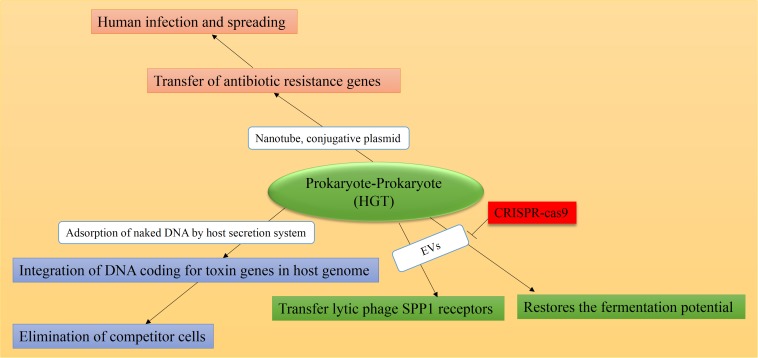
Causes and consequences of prokaryote-prokaryote HGT. Mediators are shown in white boxes and consequences are shown in colored boxes. As a natural defense mechanism in some bacteria, the CRISPR-Cas9 system can overcome horizontal transfer of genetic material, mediated by EVs and/or plasmids. HGT consequence in all cases is the emergence of resistant bacteria strains and the spread of human-related infections. Data obtained from Klieve et al. (2005), Babić et al. (2008), Husnik et al. (2013), Marraffini and Sontheimer (2008), Dubey and Ben-Yehuda (2011), Borgeaud et al. (2015), Cooper et al. (2017), Tzipilevich et al. (2017), Price et al. (2019).

A glimpse into HGT between bacterial species reveals the large and pervasive role of genetic exchange. The outcome of this interchange is the bacterial genome modification with the aim of obtaining new genetic traits and thereby rapidly reacting and further adapting to environmental changes (Figure 2; Wiedenbeck and Cohan, 2011; Croucher et al., 2016). The consequence of attracting naked DNA sequences is the recombination and evolution of many bacterial species. Likewise, acquiring bacterial pathogenicity islands (PAIs) can convert otherwise harmless species into potential pathogens (Thomas and Nielsen, 2005; Bliven and Maurelli, 2016). Needleless to say, this modification is of critical importance considering that the incorporation of ARGs into the host bacterial genomes have caused serious health problems throughout the history of mankind (Kumar et al., 2017). Generally, genetic material exchange among prokaryotes can cause genetic evolution and environmental adaptation which further promotes the acquiring of drug resistance. Importantly, both environmental adaptation and evolution are key factors in acquiring resistance to anti-microbial agents. Several mechanisms are described in anti-microbial drug resistance including antibiotic efflux pump, antibiotic degradation, enzyme inactivation, target modification, and decreased uptake, among others (Peterson and Kaur, 2018). Microbes are known to transfer the resistance gene to each other quickly; thus, the property of the antibiotic resistance spreads between the strains. The environment, as the main source of MGEs, is involved in the transfer of resistance genes (Knöppel et al., 2017). In general, the occurrence of chromosomal mutations and horizontal transfer of genetic material of antibiotic resistance are two major ways of innovation, evolution, and gene sequence variation among organisms, especially bacteria.

Horizontal gene transfer plays a substantial role in progressing drug-resistant microbes and the transfer of toxicity genes. It seems that among other HGT methods, the transformation mechanism rarely occurs between bacterial species in order to transfer resistance genes; however, the conjugation method that applies MGEs such as plasmids and transposons is a very efficient and relevant way for the spread of antibiotic resistance (Price et al., 2019). Results from an *in silico* study support that it is the phylogenetic relationships that determine the fitness and functional compatibility of horizontally transferred genes in new hosts. The results also showed that codon usage, GC content, and mRNA-folding energy play a minor role in the transfer of heterologous genes (Porse et al., 2018).

The resistance gene transfer between the body’s bacteria can transform commensal bacteria into antibiotic-resistant pathogenic bacteria, which are causative to human infection as well as its spreading (Paterson et al., 2016). In this line, *Enterococcus faecalis* is acknowledged for its good adaptability to the mammalian intestine. This native colonizer of the human gut harbors pheromone-responsive plasmids (PRPs) by which inter- and intraspecies transfer of ARGs can occur. For that, *E. faecalis* accounts for the majority of human enterococcal infections (Garcia-Solache and Rice, 2019). Plasmid mobilization is reported as a major mechanism for HGT in the evolution of resistant forms of *E. faecalis* which involves chromosomal transfer of the *E. faecalis* PAI containing virulence genes, antimicrobial, resistance capsule, and other traits (Manson et al., 2010). Likewise, the exchange of ARGs between bacteria is mainly caused by HGT phenomenon, where the conjugation mechanism plays the dominant role (Babić et al., 2008).

The advent of next-generation sequencing (NGS) as a high-throughput technique enabled the examination of resistomes (microbial communities) rather than a single pathogenic organism. NGS can be applied for the improved proactive detection and alleviation of evolving antibiotic resistance threats (Crofts et al., 2017). Using whole-genome sequencing (WGS), the effect of horizontally transferred ARGs on the elimination of the recipient bacteria sensitivity to antibiotics between enteric pathogens is studied. Data showed that the antimicrobial resistance (AMR) pattern of the 654 enteric bacteria pathogens from six representative genera was encoding resistance against 22 antibiotics from nine distinct drug classes. The presence of multiple MGEs and HGT were confirmed using WGS in the six extensive drug-resistant enteric pathogens (Kumar et al., 2017).

Some hosts can effectively defend against external DNA, such that the CRISPR system in some bacteria can be utilized to elicit immunity against phages. More than a decade ago, Marraffini and Sontheimer (2008) showed that CRISPR loci counteract ARGs’ spread among bacterial species. They showed that fluxing methicillin and vancomycin-resistant genes from *Staphylococcus aureus* into two *Staphylococcus epidermidis* strains, the strain lacking CRISPR (*S. epidermidis* ATCC 12228) acquired the transferred genes, whereas the strain containing CRISPR (*S. epidermidis* RP62a) sequences resisted against the acquisition of transmitted genes. Similarly, CRISPR loci limit the spread of antibiotic resistance in pathogenic bacteria as it can block multiple routes of HGT, including phage transduction, transformation, or conjugation (Marraffini and Sontheimer, 2008).

*Staphylococcus aureus* is a known infective bacterium with clinical importance, as it is almost resistant to all antibiotics. N315 and Mu50 strains of *S. aureus* are resistant to methicillin and vancomycin, respectively. A genome sequence survey of methicillin-resistant *S. aureus* (MRSA) and vancomycin-resistant *S. aureus* (VRSA) revealed significant levels of HGT and complexity in the genome. Studies show that 26 and 28 MGEs transmit the ARGs to N315 and Mu50, respectively (Kuroda et al., 2001; Lepuschitz et al., 2018).

The Gram-negative bacterium, *Vibrio cholerae* is known as a pathogen that conveys its toxins to nearby cells by the type VI secretion system (T6SS), and thus DNA is directly integrated into the genome of the bacterium. The *V. cholerae* spreads pathogenicity with the acquisition of resistance genes, as well (Borgeaud et al., 2015). Experimental studies conducted by Seitz et al. (2014) revealed the effective role of ComEA in absorbing and receiving DNA in *V. cholerae* cells.

*Streptococcus pneumoniae* is also considered to be another invasive bacterium. To survive and dominate under competent conditions, *S. pneumoniae* benefit from horizontal DNA transfer (HDT), as it injects toxins to kill sister/nearby cells and then extract their genomic content (Kirkup, 2017). Traces of HGT can be tracked in the pathogenesis of *Acinetobacter baumannii* with high mortality (rate of 19–54%) and high rates (>60%) of multidrug-resistance (MDR). A study reported a marked decrease of *Escherichia coli* bacteria due to the killing and receiving of their ARGs by *Acinetobacter baylyi* via HGT mechanism. *A. baylyi* as a predator lyses *E. coli* as prey by the T6SS system (Cooper et al., 2017).

Apart from the role of conjugative plasmids as HGT mediators discussed so far, Dubey and Ben-Yehuda (2011) reported that *Bacillus subtilis* transfer ARGs horizontally between the same strain of bacteria, and into the nearby *S. aureus* and *E. coli* genomes via connector nanotubes (Dubey and Ben-Yehuda, 2011).

Moreover, EVs are another effective player in the exchange of bacterial genes and transmission of infection between interspecies and intraspecies (Liu et al., 2018). Accordingly, Klieve et al. (2005) demonstrated that acquiring the genetic material through EVs restores the fermentation potential of *Ruminococcus* spp. strain YE71 to digest plant cellulose.

Likely, MVs are involved in the transient transfer of lytic phage SPP1 receptors from phage-sensitive *Bacillus Subtilis* bacteria to the SPP1-negative resistant-bacteria. For bacterial infection, phage has to be first attached to the surface of bacteria. This is mediated by the expression of extracellular phage receptors, e.g., SPP1, on the bacteria envelope. Co-culture of resistant and susceptible cells was shown to lead to the horizontal transfer of SPP1 gene, rendering phage adsorption, infection, and subsequent lysing of otherwise resistant bacteria. The results were confirmed by direct visualization of fluorescently tagged adsorbed phages on fluorescently tagged resistant cells (Ofir and Sorek, 2017; Tzipilevich et al., 2017).

### Prokaryote-Eukaryote Genetic Exchange

**FIGURE 3:**
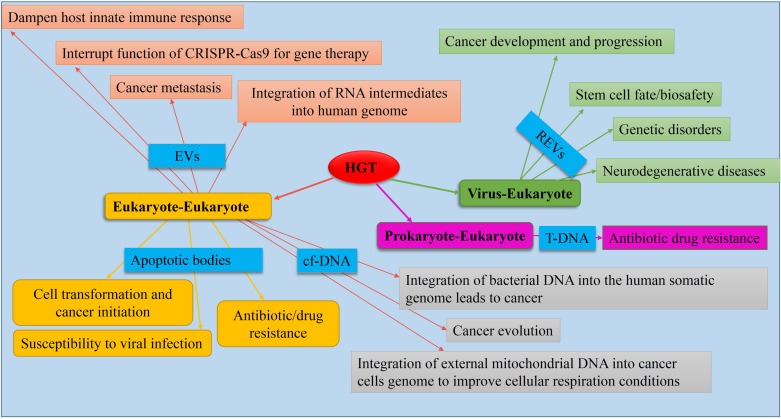
Causes and consequences of HGT in eukaryotes. Mediators (causes) are shown in blue boxes. Data obtained from de la Taille et al. (1999), Holmgren et al. (1999), Bergsmedh et al. (2001), Fronzes et al. (2009), Trejo-Becerril et al. (2012), Berridge et al. (2015), Hancks and Kazazian (2016), Lacroix and Citovsky (2016), Nishida-Aoki et al. (2017), Robinson et al. (2017), Gao et al. (2018), Jang et al. (2019), Kawamura et al. (2019), Ono et al. (2019), Schumann et al. (2019).

Evidence indicates that eukaryotic genomes are also affected by HGT, although less frequently than HGT among bacterial species (Keeling and Palmer, 2008). In this section, we evaluate the consequences of information exchange between different kingdoms in the context of evolution, adaptation, and development of AMR as well as the promotion of various health-related diseases in humans (Figure 3).

#### The Evolution of New Traits

The tardigrade, also called the water bear, is a microscopic eight-legged creature. It is of particular interest because of its capability to tolerate extraordinarily hard conditions and high stresses. In 2015, Thomas with his research team surveyed and revealed the genome sequence of this animal. Their findings showed that tardigrades have obtained about one-sixth (roughly 17.5%) of their genes from bacteria, plants, fungi, and archaea. This was an unexpected finding since almost half of that amount was the maximum known amount of possible HGT up to that moment. Foreign genes have been entered into the tardigrades’ genome over the years and have made changes in this animal’s genome composition to be able to better resist tensions. As bacteria have survived in difficult conditions for years, even decades, the researchers found that the acquisition of foreign DNA by other organisms also plays an important role in the high ability to withstand particular conditions (Boothby et al., 2015; Hashimoto et al., 2016).

Another interesting example demonstrating the role of HGT in the evolution of new traits was the recognition of mitochondria-deficient microorganisms. It was commonly believed that all eukaryotic cells are required to have a mitochondrial organelle to survive, however, in 2016 with genome sequencing of an anaerobic eukaryotic microorganism, *Monocercomonoides* sp., Karnkowska et al. (2016) discovered that the evolution of this microbial unicell has been in a way that means it generally lacks mitochondrial proteins and their encoding genes. Further analysis revealed that *Monocercomonoides* sp. replaces the cytosolic sulfur mobilization system (SUF) to provide Fe-S clusters, achieved by the horizontal transfer of the mitochondrial iron-sulfur cluster system from other bacteria (Karnkowska et al., 2016.)

Numerous studies demonstrate that the evolution of asexual reproduction in bdelloid rotifers has undergone HGT over the years in order to adapt to microenvironmental changes. Horizontal gene acquisition is one of the ways to maintain intact genes in bdelloids. Bdelloid rotifers are microscopic multicellular animals that exist in aquatic environments. These microorganisms are resistant to harsh conditions and are even able to survive in a completely dry conditions lacking all water. Under these conditions, the double-stranded DNA breaks down, and, after exposure to enough water, foreign DNA is acquired horizontally from prokaryotes and even in some cases from other rotifers; in this manner, these microorganisms can repair their chromosomes. Despite the occurrence of ameiotic reproduction in bdelloids, changes and diversity in genes and ramifications in the rotifer species are feasible through the phenomenon of HGT. Subtelomeric sequences of the bdelloids chromosomes indicate a high volume of external and non-metazoan genetic material. Phylogenetic analysis of Adineta vaga genomes as a bdelloid describes the existence of more than 8% of transferred foreign genes laterally in their genome (Boschetti et al., 2012; Flot et al., 2013; Eyres et al., 2015).

Moreover, the symbiosis of *Planococcus citri* mealybug and Tremblaya princeps, beside its endosymbiont, Moranella endobia is considered as a complex form of HGT. The mealybug sucks the plants’ juice, but under unpleasant conditions and nutrients shortage, the transmitted genes which account for 22 genes from both symbiotic bacteria and other bacteria provide essential nutrients to their insect hosts. Since the Tremblaya has been affected by the degeneration process over years of evolution, its correlation with Moranella compensates for the lost genes which are mostly involved in essential amino acid synthesis and metabolic functions (Husnik et al., 2013; Husnik and McCutcheon, 2016; Szabó et al., 2017).

#### Environmental Adaptation

The way organisms can survive in arsenic-rich environments is a clear example of cross-kingdom HGT that enables extremophilic eukaryote to adapt to life, as it has horizontally received ArsM As(III) SAM methyltransferase form bacteria or archaea. Thus, the transfer and diffusion of arsM genes horizontally from bacteria to other kingdoms have made it possible to resist the toxicity of this metal. Arsenite [As(III)] is an inorganic trivalent arsenic which is converted into a more toxic form called methylarsenite [MAs(III)] during the methylation reaction by the arsM gene. It has been observed that in addition to the arsM genes that exhibit preserved S-adenosylmethionine-binding motifs to transport methyl to As(III), three preserved cysteine residues of ArsM protein have also been detected in positions Cys72, Cys174, and Cys224 in the thermophilic algae *Cyanidioschyzon merolae sp. 5508* (CmArsM). Further, the ArsH gene converts MAs (III) into the less toxic methylarsenate [MAs(V)] through oxidation (Chen et al., 2017).

Likewise, *Helicobacter pylori*, the pathogenic gastric bacterium, uses the transition of nickel metal to enter and survive in the stomach. It acquires nickel from the external environment through NiuBDE and NixA transport systems. The common ancestor of *H. pylori* species in the stomach is responsible for obtaining NiuBDE and NixA genes during evolution by HGT. Nickel is the cofactor for urease enzyme. Urease generates carbon dioxide (CO_2_) and ammonia (NH3) buffers via urea hydrolysis which can neutralize the acidic conditions of the stomach, and subsequently adapt bacteria to the gastric acidic environment (Fischer F. et al., 2016).

*Hypothenemus hampei*, also known as a coffee berry borer, is a serious coffee pest that causes a lot of damage annually to the coffee industry around the world. The invasive beetle obtains this feature through the HhMAN1 gene gain from its gut bacteria. The encoded mannanase enzyme as a secretory protein provides the insect’s food and the ability to absorb carbohydrate from coffee beans via hydrolysis of the stored polysaccharide in coffee seeds, galactomannan. The absence of HhMAN1 in insects and the similarity of *H. hampei* genome sequences with the bacterial sequences strengthens the possibility of horizontal transfer of mannanase gene from intestinal bacteria to *H. hampei* causing the beetle to adapt to a special environment (Acuña et al., 2012).

Mite and lepidoptera species acquire cysteine synthase (CYS) and β-cyanoalanine synthase (CAS) genes to resist host plants’ cyanide poisoning by cyanogenic glucosides (CNGs), which have a defensive role against herbivores by releasing hydrogen cyanide (HCN). The expression of horizontally transmitted genes from bacteria to the arthropods’ genomes leads to HCN detoxification (Zagrobelny et al., 2004; Wybouw et al., 2014).

#### Transfer of Drug Resistance Genes

The presence of antibiotic-resistant bacteria (ARB) or ARGs in the environment and their subsequent acquisition by clinically important microorganisms, such as plants, is a serious health-related issue. Soil fungi is shown to cause the transfer and distribution of bacteria in different parts of the soil. Naturally, fungi can spread over distances and can enrich bacteria by harboring cryptic- and nutrition-assisting plasmids in their mycosphere. Moreover, soil fungi as ARGs’ carriers facilitate HGT and increase the chance of distributing ARGs from bacteria to humans. Subsequently, the wave of ARGs spread is formed in humans with uncontrolled use of antibiotics, and also via the food chain. Irrigation of plants with wastewater and animal manure contaminated with resistant genes are also very effective in the dissemination of ARGs via changing diversity and abundance of bacteria hosting the resistance determinants (Pärnänen et al., 2016; Nazir et al., 2017).

*Agrobacterium tumefaciens* is a clear example of HGT from bacteria to eukaryotes. This Gram-negative bacterium is able to transfer and merge DNA into plant genomes, causing crown-gall disease. To this, the physical attachment of Agrobacterium to the cell wall of the plant is required to insert its T-DNA. The transfer of these DNA macromolecules to the cells is possible through the type IV secretion systems (T4SSs). This secretion system has the ability to spread the antibiotic resistance by the transfer of plasmids. Later, this microorganism was exploited as a useful transfer system for genetic engineering. In this way, bacteria are capable of changing and evolving eukaryotes genomes mediated by HGT (Fronzes et al., 2009; Lacroix and Citovsky, 2016).

### Virus-Eukaryote Genetic Exchange

As a subset of TEs, endogenous retroviruses (ERVs) have been integrated into the human genome by retroviral infections of the germline cells of our ancestors where, over a long period of evolutionary time, each REV is dispersed across the genome in hundreds to thousands of repetitive copies (Wallau et al., 2018). Nonetheless, transcriptome analyses of somatic tissues surrounding tumor cells indicated co-expression of genes with TEs, extending the idea that transposon is expressed in both somatic and germ-line cells (Chung et al., 2019).

Co-opted by the host during evolution and having the capability of self-duplication and mobilization, ERVs provide a massive pool of autonomous gene regulatory modules, indispensable for normal regulation of genes and gene networks. Examples include p53-mediated regulation (Wang et al., 2007), species-specific regulatory networks (Kunarso et al., 2010), pluripotency regulation in stem cells (Lu et al., 2014; Wang et al., 2014), interferon response (Chuong et al., 2016), and activity of tissue-specific enhancers (Jacques et al., 2013; Xie et al., 2013).

The only TEs capable of mobilizing in the human genome belong to a group member of the non-LTR retrotransposons, which include autonomous long interspersed class 1 elements (LINE-1 or L1) and non-autonomous short interspersed elements (SINEs), such as Alu and SVA [SINE- variable number of tandem repeats (VNTR) –Alu elements]. Adopting a “copy-and-paste” mechanism, LINE-1 or L1 not only mobilize its own RNA to new sites in the genome, but also retrotranspose other RNAs including Alu, SVA, and cellular RNAs (Romanish et al., 2010).

Accordingly, besides their wide implication in normal gene regulation processes and their application as useful tools for genome editing and gene therapy purposes, on the dark side, the high rate of transposition rate (Romanish et al., 2010) can negatively affect important regulatory networks, resulting in pathologic conditions. It has been reported that∼124 LINE-1-mediated insertions can cause specific genetic diseases (Hancks and Kazazian, 2016), different types of cancer (Babaian and Mager, 2016), and neurodegenerative diseases (Tam et al., 2019). They also adversely affect the prospect of stem cell-based therapies (Schumann et al., 2019).

#### ERVs and Cancer

In cancers, hypomethylation (Ross et al., 2010) and transcriptional overexpression of ERVs and L1s are frequent. During onco-exaptation, silent TEs can be epigenetically reactivated to promote cancer. To date, analysis of the prevalence of TE onco-exaptation events across 7,769 tumors and 625 normal datasets from 15 cancer types resulted in the identification of 106 oncogenes out of 129 TE cryptic promoter-activation events across 3,864 tumors. Further interrogation of the AluJb-LIN28B candidate revealed the sufficiency and necessity of a TE for oncogene activation (Jang et al., 2019). Furthermore, activation of the hypoxia-inducible transcription factor (HIF), which is a characteristic feature of malignant renal cell carcinoma (RCC), is shown to be related to HIF-dependent reactivation of dormant promoters that are embedded within ERV LTR at the transcriptional start site of a long non-coding RNA (PSOR1C3) gene upstream of POU5F1. Downstream of HIF, POU5F1 (OCT4) acts as a potent stem cell transcription factor (Siebenthall et al., 2019). Likewise, derepresssion of an ERV, LOR1a LTR upstream of IRF5 transcription factor interferon regulatory factor 5 (IRF5), forms LTR-IRF5 chimeric transcripts, which derives IRF5 expression as a feature of malignancy in hodgkin lymphoma (Babaian et al., 2016).

A study on genomic instability pathways arising from retrotransposon insertions in colorectal cancer (CRC) revealed highly variable retrotransposon activity among tumors and identified recurrent insertions in 15 known cancer genes. Insertions in APC as a tumor-initiating event was found in 1% of the 202 cases. Clinically, the high number of insertions are independently associated with poor disease-specific survival (Cajuso et al., 2019).

#### TEs in Stem Cell-Based Therapies

Human stem cells are indispensable for most basic, clinical, and translational research, such as gene therapy and regenerative medicine. However, they are wider in the clinic and are still limited by their potential risks due to genomic instability and tumorigenesis concerns (Schumann et al., 2019).

Owing to their self (non-self)-mobilizing potential, ERs are used as genetic engineering toolkits to introduce foreign DNA (transgene) to desired genomic regions in target cells. Moreover, L1 has a role in the normal development of the embryo. After fertilization, global chromatin accessibility at the beginning of development by L1 transcriptional activation is important for normal embryonic development (Jachowicz et al., 2017). Also, the L1 RNA is required for repression of the transcriptional program specific to the 2-cell embryo and promotes exit from the 2-cell stage (Percharde et al., 2018). Genotoxicity concerns for retrotransposon-mediated gene transfer are contradictory and reviewed in detail elsewhere (Schumann et al., 2019).

#### Human Genetic Disorders

Random L1 mobilization in our genome can result in numerous disorders. These disorders are caused by the mutagenic activity of non-LTR retrotransposons, with a rate of 1–250 pathogenic human mutations due to L1-mediated retrotransposition events. Mutations are detected in 5% of newborns and more than 120 known human diseases are related to insertions of L1s, Alus and SVAs (Cordaux et al., 2006; Ewing and Kazazian, 2010; Hancks and Kazazian, 2012, 2016). Besides cancer, as a typical example of aberrant mutational/epigenetic changes, L1 insertions are involved in the pathogenesis of several genetic disorders (Payer and Burns, 2019). Some of them arise from the addition/deletion of tri-nucleotide pairs that affect the expression of the corresponding gene as seen for the expansion of a hexameric repeat within an SVA retrotransposon in TATA-binding protein-associated factor-1 (TAF1) in X-linked dystonia-parkinsonism (Bragg et al., 2017). Others include Huntington’s disease (HD) (Goldberg et al., 1993), hemophilia A (L1) (Kazazian et al., 1988), and cystic fibrosis (Alu) (Chen et al., 2008). A complete list of ER-related human diseases can be found in Hancks and Kazazian (2016).

#### Neurological Diseases

Differential TE activity in neurological diseases is detected by an increase in TE-associated RNAs, cDNAs, and proteins in patient samples. Although specific TE-derived products can result in different consequences, there is a common pathogenic reaction, known as general inflammatory response. This (innate) inflammatory responds to cDNAs (by ERV-mediated reverse transcription), dsRNAs (by TE-mediated transcription), or proteins as demonstrated in Aicardi-Goutieres Syndrome (AGS), Amyotrophic Lateral Sclerosis (ALS), Multiple Sclerosis (MS), and Alzheimer’s Disease (AD) (Tam et al., 2019). In MS and AGS, ERV proteins and TE nucleic acids can derive innate immune cells-derived inflammation pathways. In AD and ALS, TEs-related pathogenic effects are localized to either hippocampal or cortical neurons (in AD) and motor neurons (in ALS). In addition, expression of envelope proteins from the HERVK and HERVW class have been shown to be neurotoxic and implicated in ALS and MS, respectively (Saleh et al., 2019).

### Eukaryote-Eukaryote Genetic Exchange

In this section, we review the implication of newly emerged HGT tools adopted by eukaryotic cells to promote the emergence of beneficial traits as well as evolving numerous human-related maladies, in particular cancer.

#### Eukaryotic Evolution

Plants, in particular parasitic plants, accept a large quantity of genes horizontally. In parasitic plants, such a genome exchange is partly due to the food dependency to their host. Parasitic plants penetrate the tissues of donor plants by their haustorium to intake nutrients along with their genetic material. Thus, HGT monitoring will help to control the generation and function of parasitic plants, which is one of the most important agricultural problems in the world. DNA transferred horizontally can lead to the adaptive evolution of the recipient plants. Results from transcriptome analysis of three parasitic members of Orobanchaceae (*Triphysaria versicolor*, *Striga hermonthica*, and *Phelipanche aegyptiaca*) and a non-parasitic member (Lindenbergia) showed 52 DNA non-sexual transfers between the parasite members of Orobanchaceae and their hosts. These data show that the greater dependence of parasitic plants on their hosts might increase the chance for HGT, and HGT occurs more significantly in heterotrophic plants than non-parasitic plants (Yang et al., 2016). For instance, the existence of a common gene called ShContig9483 between the Striga hermonthica parasitic plant and its host, Sorghum bicolor, indicates that one sorghum’s gene has been entered into the parasite plant’s DNA by HGT (Yoshida et al., 2010). Moreover, the transfer of two active hAT DNA transposon elements from Brassicaceae to Phelipanche and Orobanche suggested that the Broomrape as a common ancestor has diverged to these parasitic plants following HGT (Sun et al., 2016). Another example is acquiring host plant mRNA (albumin 1 KNOTTIN-like protein gene) by lespedeza dodder (Roney et al., 2007) by HGT from legumes to Cuscuta members and Phelipanche aegyptiaca (Zhang et al., 2013). Entering a large number of genes into the *Amborella trichopoda* mitochondrial genome by angiosperm, mosses and green algae as donors (Rice et al., 2013) are examples of transduction and translocation of host plants genetic material to parasites.

The issue of the transmittance of mitochondrial cytochrome c oxidase I (COX1) group I introns into the Araceae family has already been investigated by Cusimano et al. (2007). They demonstrated that fungi donate the introns into these flowering plants to promote the evolution of angiosperms.

Similarly, phylogenetic studies demonstrate the distribution of the hAT superfamily DNA transposons horizontally between Drosophila species (Schaack et al., 2010; Rossato et al., 2014). A paper by Ivancevic et al. (2018) reported the occurrence of horizontal transfer in two retrotransposons, LINE-1 (L1) and Bovine-B (BovB), in the mammals’ genome. Their research team traced and scrutinized these two foreign genes in a large number of eukaryotes genomes and showed evidence that they have identified for the first time the horizontal transmission in L1 elements. Also, they stated that plenty of lateral BovB elements are transferred between various species (Ivancevic et al., 2018).

#### Cancer Evolution

Recent studies have uncoupled new mediators of HGT by which cancer cells can crosstalk and send signals to transform distant normal/cancer cells. This is important as these events are actively involved in cancer progression and metastasis outbreak. In this section, we discuss the implication of HGT mediators including EVs (exosomes), apoptotic bodies, and cfDNA in the emergence and progression of cancer, as well as the development of drug resistance.

#### Apoptotic Bodies

Evidence suggests that apoptotic cells derived from dying cells (tumor cells) can laterally transfer complex information to other cells, as they are shown to carry various cargoes including DNA, RNA, and protein. Further, changes in the outer membrane of apoptotic cells can induce neighboring cells to become phagocytic (de la Taille et al., 1999), thus apoptotic cells can be engulfed by normal adjacent cells, causing the exchange of genetic information in the context of adopting new traits, e.g., oncogenic transformation, or drug resistance in the receipt cells. The consequence of apoptotic-mediated information transfer is not always pathologic, as in the case of bone remodeling which occurs each 2-weeks, a large number of apoptotic bodies are produced by mature osteoclasts. These apoptotic bodies induce osteogenic differentiation via RANKL-mediated reverse signaling (Ma et al., 2019).

Earlier experiments indicated that oncogenic DNA can be transferred to cells by phagocytosis of apoptotic bodies. Results showed that apoptotic bodies derived from EBV-carrying B lymphocytes when cocultured with other cells (e.g., fibroblasts) result in the expression of the EBV-encoded genes EBER and EBNA1 in the recipient cells at a high frequency. Thus, HGT mediated by apoptotic cells can be a route for infection of cells that lack the receptor for the virus (Holmgren et al., 1999).

A similar experiment is reported where DNA coding for the ARGs (hygr-r) can be transferred by apoptotic prostate cells to the Neomycin-resistant and bcl-2 overexpressing LNCaP prostate cells resulting in dual-antibiotic resistant prostate cancer cell clones (de la Taille et al., 1999).

Furthermore, the transfer of viral DNA after uptake of apoptotic bodies is confirmed to be a driver of HIV DNA toward immune cells lacking the CD4 receptor for virus entry *in vivo* (Spetz et al., 1999).

Uptake of apoptotic bodies derived from H-rasV12- and human c-myc-transfected rat fibroblasts by p53-negative murine phagocytic cells results in loss of contact inhibition *in vitro*, aneuploidy, and the accumulation of the genetic changes that spark tumor formation *in vivo* (Bergsmedh et al., 2001).

With regard to the role of donor apoptotic cells in the regulation of HDT, it was shown that the inhibition of DNA fragmentation factor/caspase-activated DNase (DFF/CAD) can reduce the competency of cells to serve as good donors. It should be noted that DFF/CAD is the endonuclease responsible for DNA fragmentation during apoptosis (Yan et al., 2006). Further studies indicated that besides DNase II, the activity of Chk2, p53, and p21 signaling pathways can counteract propagation of apoptotic bodies-derived oncogenic DNA, thus it forms a genetic block against transformation and malignant phenotype (Bergsmedh et al., 2006).

#### Circulating Cell-Free DNA

Besides apoptotic bodies, cfDNA is another precious tool with an implication in diagnosis, prognosis, and monitoring of cancer. cfDNA can originate from apoptotic cells and necrotic cells as well as from active cellular secretion. The size of the DNA derived from apoptotic degradation fairly extends to ∼166 bp composed of one nucleosome (147 bp). However, cancer cell-derived cfDNA is shown with a smaller size (∼90 bp) compared to normal cells. Also, larger DNA up to 10,000 bp with a necrotic cell origin is detected in cancer patients (Jahr et al., 2001). Mitochondrial DNA (mtDNA) is another example of cfDNA released into blood circulation during cellular clearance or repair processes. Another cfDNA with a different size of 1000 bp up to 20,000 bp for chromosomal circular DNA is also reported (Bronkhorst et al., 2019).

Various experiments have documented the oncogenic potential of cfDNA on normal cells. For one, experiments on colon cancer transformation indicated that the supernatant of SW480 human cancer cells and plasma-derived from patients with colon cancer can be transferred laterally to the healthy cells and transform cultured murine NIH-3T3 cells ontogenetically, as validated by quantification of K-ras-mutated sequences, p53 sequences, and beta-globin-encoding sequences (García-Olmo et al., 2010; Trejo-Becerril et al., 2012). Thus, recipient cell genetic alteration with the potential to derive tumorigenicity and development of tumors are among the most serious subjects related to HDT (Trejo-Becerril et al., 2012).

Viral DNA integration into the human genome is an accepted fact, however, new studies indicated integration of bacterial DNA into the human somatic genome. This observation has now prompted a new hypothesis that the origin of cancer cells are bacteria. It equally questions the source of primary cancer cells (PCCs) and secondary metastatic cancer cells (SCCs) which are proposed to be originated from the senescent normal cells and cancer cells, respectively. Indeed, there is a resemblance between the nascent PCCs/SCCs that are small and organelle-less resembling bacteria with that of cyanobacterium TDX16 development and transition. TDX16 acquires algal host DNA which turns it into alga TDX16-DE by *de nevo* biogenesis of organelles. This observation suggests bacteria as the mighty origin of cancer cells (Dong and Xing, 2018).

Additionally, there is clinical evidence that bacterial DNA integrates into the human somatic genome through an RNA intermediate, as insertion of *Acinetobacter* and *Pseudomonas* genetic materials into human genome by HGT is validated which is causative to the development of acute myeloid leukemia (AML) and stomach adenocarcinoma, respectively (Riley et al., 2013; Robinson et al., 2013, 2017). Also, data analysis of public cancer genome sequence data confirmed an association between the presence of mycobacterium tuberculosis complex in the glioblastoma multiforme (GBM) and ovarian serous cystadenocarcinoma (OV) samples, as well as the presence of *Ralstonia* spp. in AML samples (Robinson et al., 2017).

One of the most critical factors in tumor formation and development is mitochondrial respiration affecting the effective extension of metastasis. Notably, the results of the conducted studies in this field point to the fact that tumor cells receive horizontally the mtDNA from other cells of the body to improve the cellular respiration conditions (Berridge et al., 2015; Dong et al., 2017).

#### Exosome

As another intriguing mechanism of HGT, the biological relevance of EVs in intercellular communication has been well established, as they can carry different types of biomaterials including proteins, RNA (mRNAs and microRNAs) (Cai et al., 2013), and DNA [gene fragment, genomic DNA (gDNA)] (Fischer S. et al., 2016) as main cargo. Cargo can be integrated on the outer or inner layer of the vesicle phospholipid membrane through anchoring proteins or can be packed inside the vesicle.

Besides normal cells, apoptotic cell-derived extracellular vesicles (ApoEVs) are also described as containing nuclear materials and mitochondria and presenting cell-specific markers on the surface, which enable tracking of the cell of origin (Jiang et al., 2017; Poon et al., 2019). Interestingly, EVs are shown to mediate the horizontal transfer and integration of RNA intermediates into the human genome. To this, human cancer cells were transfected with an expression construct containing a retrotransposition-competent human L1 tagged with a reporter gene. Further analysis of isolated EVs showed enrichment of L1-derived reporter RNA transcripts from cancer cells expressing active L1 retrotransposition (Kawamura et al., 2019).

Discovering the tumor-derived exosomes in cancerous mice revealed the releasing of epidermal growth factor receptor (EGFR) in host macrophages which cause a decrease in interferon-1 gene expression, and thereby the immune response is reduced against viral infection (Gao et al., 2018). Thus, tumor cells can transfer activated EGFR through EVs to the host macrophage to dampen innate immunity response, rendering the host immunocompromised.

Initial experiments validating genomic DNA transfer of a model gene by exosomes comes from the study in which a lentiviral vector encoding Arabidopsis thaliana-DNA (A.t.-DNA) was transduced to bone marrow-derived mesenchymal stromal cells (BM-hMSC). It was previously shown that EV released from human BM-hMSC has a high content of high-molecular DNA. Further quantification and verification of PCR-products from the recipient cell suggested stable integration of A.t.-DNA for two weeks (Fischer S. et al., 2016).

Complex exchange of genetic information is evidenced in the pathogenesis of cancer. Indeed, the BCR/ABL hybrid gene can be transferred from K562 EVs to neutrophils/HEKs, which further decrease their phagocytic activity *in vitro*. Also, the transfer of K562 EVs in immunodeficient mice results in chronic myeloid leukemia (CML) (Cai et al., 2013; Cai et al., 2014). Likewise, microRNA-containing EVs derived from the sera of patients with colorectal cancer as well as HT29 colon cancer cells can induce the transformation of fibroblasts into colon carcinoma cells (Abdouh et al., 2019). In this view, a therapeutic strategy can be envisioned by targeting cancer-derived EVs. Such that breast cancer metastases to the lungs, lymph nodes, and thoracic cavity can be controlled using human-specific anti-CD9 or anti-CD63 antibodies which target CD9/CD63 receptor on the surface of EVs. Finally, depletion of EVs, as evidenced by their preferential internalization by macrophages, was capable of reducing metastatic burden in a human breast cancer xenograft mouse model (Nishida-Aoki et al., 2017).

Interestingly, HGT can affect the therapeutic potential of the leading-edge technology CRISPR-Cas9 system. A drawback of gene manipulation by the CRISPR-Cas9 system is unintentional integration of DNA sequences derived from retrotransposons, genomic DNA, mRNA, and vectors which are captured at double-strand breaks (DSBs) sites introduced by CRISPR-Cas9. Exosome-mediated HGT during DSB repair can negatively affect the therapeutic efficiency of CRISPR-Cas9 technology (Ono et al., 2019).

As EVs are present in all fluids of living animals, exosome-mediated HGT is the driving force behind mammalian genome evolution which can be translated into cancer development as well as adaptation and drug resistance (Baghban et al., 2020).

Engineered exosomes that display tumor-specific targeting receptors (e.g., Her2) can be loaded with chemotherapeutic (Doxorubicin) (Gomari et al., 2018) or theranostics agents [sinoporphyrin sodium (Liu et al., 2019)] for selective targeting and delivery of therapeutics to cancer cells. In the sense of gene therapy, siRNA can be delivered to breast cancer cells by exosomes, which resulted in 70% down regulation of the target gene (TPD52) *in vitro* (Limoni et al., 2019).

In an interesting study, metastatic-Trap (m-tarp) was manufactured using exosomes embedded on a three-dimensional platform as a trap (favorable secondary site) to lure metastatic cells. For this, exosomes purified from the ascitic fluid of ovarian cancer patients were validated as intermediaries of tumor cell attachment. Transplantation of the device into the peritoneal cavity of mice resulted in the capturing of ovarian metastatic cells and significantly improved survival outcomes by disrupting the crosstalk between metastatic cells and their environment (de la Fuente et al., 2015).

On the bright side, the potential of exosomes as natural and non-toxic agents (vectors) to induce long-term changes in the receipt cells was further translated into the gene therapy as an alternative to the time-consuming and laborious gene cloning as well as for targeted (functionalized exosomes) and controlled drug delivery.

#### Cellular Organelles

What’s more interesting is the diversity of biomaterials that can be transferred inter/intraspecies encompassing genes, genomes, proteomes, and even organelles.

Indeed, the evidence for HGT related to the transfer of mitochondria has changed the previously accepted paradigms regarding the role of anaerobic glycolysis in cancer cell metabolism. Although glycolytic metabolism is recognized as the key hallmark of many cancer cells, tumor cells also need oxidative phosphorylation (OXPHOS) to meet their pathophysiological demands. On the contrary to the previous fact suggested by Warburg that defects in mitochondria are causative to cancer initiation, some cancer cells retain OXPHOS capacity with no clear respiratory defects (Frezza and Gottlieb, 2009; Jose et al., 2011). Furthermore, inhibiting glycolysis may restore higher rates of OXPHOS in neoplastic cells and spark cancer growth. Respiration is key for cancer cell formation, proliferation, progression, and metastasis (Viale et al., 2015). Surprisingly, cancer cells deprived of mtDNA can restore their respiration potential through a rare phenomenon which is regulated by HGT involving the transfer not of mtDNA but of whole mitochondria (Tan et al., 2015).

Results from an invaluable study revealed that mtDNA-deficient tumor display delays in tumor formation potential compared to their normal counterpart. It also showed that progression was associated with respiration recovery and acquiring mtDNA from the host. Further studies showed that efficient tumor formation, recovery of mitochondrial respiration, and mtDNA acquisition occurs via trafficking of whole mitochondria between mammalian cells *in vivo* (Dong et al., 2017). It is also shown that mitochondria exchange between leukemic cells and mesenchymal stem cells residing in the bone marrow niche increases oxidative phosphorylation of cancer cells and contributes to leukemia cells’ survival and resistance to chemotherapy (Griessinger et al., 2017). As a therapeutic strategy, mitochondria transplantation is demonstrated to improve antitumor activity and reduce chemoresistance and mitochondrial dynamics in breast cancer *in vitro* (Chang et al., 2019).

## Conclusion and Future Prospective

Life under a constant dynamic microenvironment requires specific tolls to enable micro-organisms’ adaptation to new conditions. Since the genetic remodeling of a single organism is not enough to meet this requirement, HGT has emerged as an effective approach to provide such a survival advantage.

The consequences of HGT can be for or against acquiring genetic/phenotypic changes in the recipients’ cells. The appearance of phenotypic changes can be immediate (e.g., bacterial drug resistance) or delayed (a neurodegenerative disease). In general, it is an undeniable fact that HGT occurs in/between all five kingdoms of organisms.

Horizontal gene transfer acts as a double-edged sword: although it fuels innovation and diversity in nature, now, its effects function against the survival benefit in nature, especially in humans. For one, the dawn of the post-antibiotic area is highly predicted as a consequence of HGT in the future, at a time point in which the bacteria will no longer respond to antibiotics.

Equally, in the context of other diseases, such as cancer, HGT not only promotes the development of the clonal population of cells capable of tumorgenesis, it also sparks tumor cell heterogeneity and the emergence of multi-drug resistance (Dianat-Moghadam et al., 2018; Seidi et al., 2018). Such survival advantage to tumors is mediated, partly, by the newly discovered HGT mediators in the eukaryotes. DNA from apparently dead tumor cells, called apoptotic bodies, can be transferred to living tumor cells, as naked, in complex with other biomaterials or being packed into the EVs. Organotypic metastasis, which refers to the tendency of tumors to seed in selective and preferred organs, has recently been shown to be regulated by exosomes. Indeed, the expression of specific surface receptors on exosomes is shown to prepare secondary organs for tumor metastasis. And that disrupting these interactions can selectively avoid metastatic outbreaks in several mice models (Hoshino et al., 2015; Guo et al., 2019).

Accordingly, the most important matter about HGT is to understand its transmission mechanism (s), which, in turn, will provide many advantages and opportunities. For example, instead of focusing on the development of new antibiotics, the horizontal transmission mechanism in bacteria will be considered as a therapeutic target. Given that the process for the development of new antibiotics is time-consuming and expensive, disruption of HGT in bacteria populations, using, for example, the CRISPR-Cas9 system, can disconnect genetic exchange among bacteria. Thus, not only can the development of antibiotic drug resistance be prevented, but inter- and intra-species transmission of virulence factors can be reduced as well. This is of paramount importance given the ever-increasing cases of acquired resistance to a wide range of routinely- and clinically used antibiotics.

From another angle, a comprehensive understanding of HGT mediators will offer a great benefit to genetic engineering. Given the high costs and low efficiency of current gene therapy tools, recognition of HGT mechanisms can lead to the development of more efficient and cheaper gene therapy methods, for treatment of postnatal genetic diseases such as those related to a specific enzyme deficiency (metabolic disorders) or even for the treatment of cancer.

Besides experimental studies, the use of recently spotted machine learning and/or systems biology approaches as robust predicting tools can help to understand the biology of HGT. That is to say, we now know that genetic fragments features with a mobility potential and characteristics of donor organism can be used as data to predict possible insertion-, location site and/or feasible recipient organisms. Thus, it can predict the recipient bacteria that will become resistant to specific antibiotics. As the target host is already predicted, a combination modality can be highly effective to monitor resistance to the newly introduced antibiotics in the clinic.

Equally, machine learning can be applied for the prediction of mobile elements movement in the human genome. Half of the human genome is composed of inserted sequences from other organisms, of which only transposons have the capability of jumping and transferring gene fragments in the eukaryotic DNA. As the role of TEs is well established in the pathogenesis of multifactorial diseases, such as cancer and neurodegenerative disease, a machine learning approach can spot the next location of jumping elements in the human genome. Once the new jumping site is recognized, then one can predict the good or bad consequence of transposon insertion. Considering the genomic variations among different individuals, the possibility for prediction of jumping patterns of the selfish DNA (TEs) among different peoples/populations can be used for personalized medicine, susceptibility prediction to a specific disease, and identification of new drug targets.

Finally, the more recent clinically important mediators of HGT among eukaryotic cells are exosomes, cfDNA, and apoptotic bodies, through which distant cancer cells can communicate together. The newly emerged cancer diagnostics, called liquid biopsy, seeks and screens patients as well as susceptible individuals’ blood samples for the presence of these clinically important signatures. Capturing these rare biomarkers by point-of-care ultrasensitive detection systems (biosensors) can provide valuable information regarding tissue of origin, disease stage, disease progression monitoring, drug response, screening, and prediction of disease resistance/relapse. Thus, it allows the clinicians to plan the most suitable drug regimens based on disease state and adopt a timely intervention to control the disease and achieve desirable therapeutic effects (Baghban et al., 2020). Liquid biopsy is still in its infancy and the technological advancements, which aim to enable single cells/droplets of biological fluids, are actively pursued across the world. Thus, horizontal transfer mediators may be the next generation point-of-care diagnostic, prognostic, and therapeutic tools to predict, cure, and prevent various human-related maladies, in particular cancer.

## Author Contributions

All authors listed have made a substantial, direct and intellectual contribution to the work, and approved it for publication.

## Conflict of Interest

The authors declare that the research was conducted in the absence of any commercial or financial relationships that could be construed as a potential conflict of interest.
